# Hybrid Exercise Program for Sarcopenia in Older Adults: The Effectiveness of Explainable Artificial Intelligence-Based Clinical Assistance in Assessing Skeletal Muscle Area

**DOI:** 10.3390/ijerph19169952

**Published:** 2022-08-12

**Authors:** Meiqi Wei, Deyu Meng, Hongzhi Guo, Shichun He, Zhibo Tian, Ziyi Wang, Guang Yang, Ziheng Wang

**Affiliations:** 1 Chinese Center of Exercise Epidemiology, Northeast Normal University, Changchun 130024, China; 2 Graduate School of Human Sciences, Waseda University, Tokorozawa 169-8050, Japan; 3 AI Group, Intelligent Lancet LLC, Sacramento, CA 95816, USA; 4 College of Physical Education and Health, Guangxi Normal University, Guilin 541006, China; 5 Advanced Research Center for Human Sciences, Waseda University, Tokorozawa 169-8050, Japan

**Keywords:** sarcopenia, older adults, exercise program, explainable artificial intelligence

## Abstract

Background: Sarcopenia is a geriatric syndrome characterized by decreased skeletal muscle mass and function with age. It is well-established that resistance exercise and Yi Jin Jing improve the skeletal muscle mass of older adults with sarcopenia. Accordingly, we designed an exercise program incorporating resistance exercise and Yi Jin Jing to increase skeletal muscle mass and reverse sarcopenia in older adults. Additionally, machine learning simulations were used to predict the sarcopenia status after the intervention. Method: This randomized controlled trial assessed the effects of sarcopenia in older adults. For 24 weeks, 90 older adults with sarcopenia were divided into intervention groups, including the Yi Jin Jing and resistance training group (YR, *n* = 30), the resistance training group (RT, *n* = 30), and the control group (CG, *n* = 30). Computed tomography (CT) scans of the abdomen were used to quantify the skeletal muscle cross-sectional area at the third lumbar vertebra (L3 SMA). Participants’ age, body mass, stature, and BMI characteristics were analyzed by one-way ANOVA and the chi-squared test for categorical data. This study explored the improvement effect of three interventions on participants’ L3 SMA, skeletal muscle density at the third lumbar vertebra (L3 SMD), skeletal muscle interstitial fat area at the third lumbar vertebra region of interest (L3 SMFA), skeletal muscle interstitial fat density at the third lumbar vertebra (L3 SMFD), relative skeletal muscle mass index (RSMI), muscle fat infiltration (MFI), and handgrip strength. Experimental data were analyzed using two-way repeated-measures ANOVA. Eleven machine learning models were trained and tested 100 times to assess the model’s performance in predicting whether sarcopenia could be reversed following the intervention. Results: There was a significant interaction in L3 SMA (*p* < 0.05), RSMI (*p* < 0.05), MFI (*p* < 0.05), and handgrip strength (*p* < 0.05). After the intervention, participants in the YR and RT groups showed significant improvements in L3 SMA, RSMI, and handgrip strength. Post hoc tests showed that the YR group (*p* < 0.05) yielded significantly better L3 SMA and RSMI than the RT group (*p* < 0.05) and CG group (*p* < 0.05) after the intervention. Compared with other models, the stacking model exhibits the best performance in terms of accuracy (85.7%) and F1 (75.3%). Conclusion: One hybrid exercise program with Yi Jin Jing and resistance exercise training can improve skeletal muscle area among older adults with sarcopenia. Accordingly, it is possible to predict whether sarcopenia can be reversed in older adults based on our stacking model.

## 1. Introduction

Millions of older people experience muscle wasting every year, which may be brought on by a number of illnesses including sarcopenia, cachexia, and muscular dystrophy. The former refers to the physiological loss of skeletal muscle mass and function with age, with up to 50% of mass being lost by the eighth decade of life [1,2]. An increasing body of evidence suggests that systemic signals cross-talk with intrinsic mechanisms that affect the quality of muscle tissue at the cellular level, including impaired signaling that leads to the reduction of protein synthesis and myofiber denervation, as well as changes in the regulation of cellular quality through the loss of regenerative potential [3,4,5]. Consequently, pathological alterations to this crucial metabolically active tissue may have serious effects on older adults, such as a severe influence on their quality of life and ability to do daily activities [6,7,8,9,10]. Moreover, as a novel prognostic indicator across cancer types [11], sarcopenia is often associated with reduced survival [11,12], toxic treatment effects [13,14,15], and poor surgical outcomes [16]. Sarcopenia combined with inflammation has been reported to nearly double the risk of death [17]. These findings underscore the critical importance of addressing the health issues of older adults with sarcopenia.

To address this key problem, several approaches have been designed in recent years. Previous studies have shown that loss of muscle plasticity during aging is associated with many factors, such as chronic low-grade inflammation [18], loss of anabolic signaling through GH/IGF-1 [19], and lower protein intake and vitamin D insufficiency [6]. Interestingly, studies have shown that exercise, hormones, specific anabolic drugs, and nutrition are beneficial against the development of sarcopenia. In this respect, much emphasis has been placed on increasing protein intake associated with lean body mass (LBM) [20,21]. However, it has been found that a protein intake needs to be implemented in accordance with the standard, beyond the recommended dietary intake (0.8 g/kg/d for the entire adult population), there is no positive effect on the increase of LBM or the enhancement of the anabolic response to testosterone [22,23]. Meanwhile, many muscle anabolic drugs such as androgens and myostatin antagonists are being developed to treat sarcopenia. Notwithstanding that significant inroads are expected in future years with the development of diagnostic strategies and therapeutic agents against sarcopenia [24], different classes of drugs can cause diverse kinds of side effects [24]. So far, the Food and Drug Administration has not approved any drugs to control muscle weakness. Drug safety is of paramount importance, and needs to be taken into account first. At the same time it needs to be addressed with new concepts and methods.

Fortunately, recent research indicates that exercise therapies have some favorable benefits on sarcopenic older people [25], and the most effective therapy for the loss of muscle mass and strength in older adults is resistance exercise training (RET) [26]. By modifying IGF-1 and its receptors, as well as the Akt/mTOR and Akt/FOXO3a signaling pathways, RET has been shown to improve autophagy activity and decrease the apoptosis of muscle cells in aged skeletal muscles [27]. Meanwhile, Chinese traditional exercise (CTE) has shown positive effects on sarcopenia. Since 2007, 13 studies based on TCE have been conducted. Among them, ten studies [28,29,30,31,32,33,34,35,36,37] involved Yi Jin Jing, an appropriate form and intensity of exercise for older adults with established physical improvement efficacy. Yi Jin Jing is a multi-component traditional Chinese mind-body therapy that combines meditation with slow, gentle, stretching muscle movements including deep diaphragmatic breathing and relaxation [38] and has twelve movements. Given that the aforementioned works of RET and CTE achieved good performance against sarcopenia among older adults, it remains unclear whether hybridized RET and TCE can increase skeletal muscle and reverse sarcopenia among older adults, warranting further studies.

In recent years, many studies have identified the need for artificial intelligence (AI), especially for healthcare applications [39,40]. Explainable artificial intelligence (XAI) represents an effort to produce human interpretable models while maintaining a high level of learning performance. An interpretable AI-based automated diagnostic platform has many advantages, and its interpretability enables the system to be more easily understood and trusted. Therefore, to facilitate the clinical applicability of the models, an easy-to-use offline XAI model was developed to predict the reversal of sarcopenia among older adults with different physical conditions, initial skeletal muscle area, and interventions.

In summary, this study integrated RET and Yi Jin Jing as an intervention to improve sarcopenia in older adults, and quantify the effects through computed tomography (CT) scans. Further, we utilized 10 classical machine learning models to predict the sarcopenia state of participants at 24 weeks based on initial handgrip strength, CT scans, and three intervention types. Based on the finding of previous studies, we hypothesized that an exercise program integrating RET and Yi Jin Jing yields better performance in increasing muscle mass and reversing sarcopenia than an RET program only.

## 2. Materials and Methods

### 2.1. Participants

**Figure 1:**
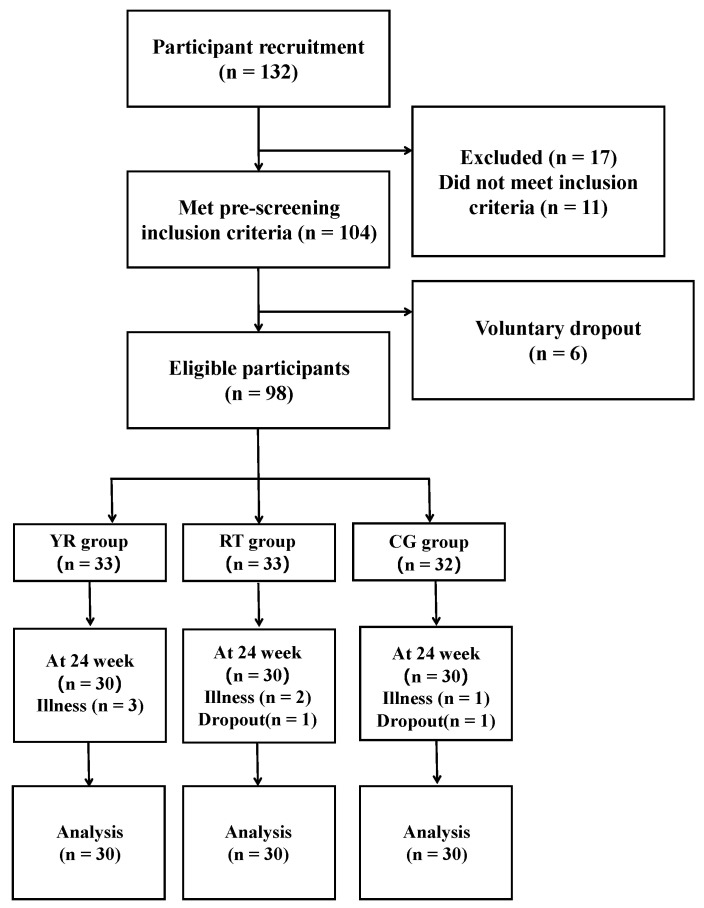
Participant flow diagram.

In this randomized controlled trial, participants were recruited through the Health Screening Center of Baishan Central Hospital and Baishan Ciming Health Screening Center in Baishan, China. Participants were screened according to the following inclusion and exclusion criteria. Inclusion criteria: (1) patients that met the Asian screening criteria for sarcopenia established by AWGS 2016 [41] and (2) aged between 60 and 75 years old. Exclusion criteria: (1) participants with respiratory failure or severe physical illness; (2) participants with neuromuscular disorders; (3) participants taking medications that have a significant impact on musculoskeletal function; (4) participants with mental disorders or neurological disorders; (5) regular participation in other training programs. Thus, the sample size was calculated based on a previous study [42], with 80% power at an alpha level of 0.01 and an effect size of 0.53, and a dropout rate of 20%. The participant flow diagram was shown in Figure 1.

### 2.2. Study Design

#### 2.2.1. Experimental Arrangement

**Figure 2:**
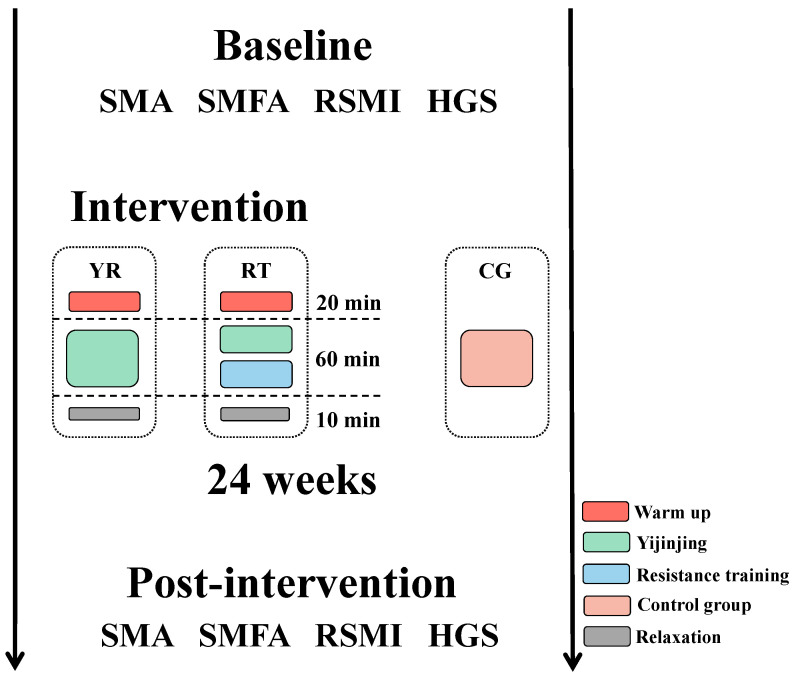
Experimental design. All participants were randomly allocated to the YR, RT, and CG intervention groups. Assessment of participant’s skeletal muscle cross-sectional area at the 3rd lumbar vertebra (L3 SMA), skeletal muscle interstitial fat area at the 3rd lumbar vertebra (L3 SMFA), relative skeletal muscle mass index (RSMI), and handgrip strength (HGS) at pre-intervention and post-intervention.

This was a double-blind, randomized controlled trial designed to determine the effects on skeletal muscle in older adult patients with sarcopenia using quantitative CT (QCT), as shown in Figure 2. Participants were recruited from the Health Screening Center of Baishan Central Hospital and Baishan Ciming Health Screening Center, Baishan, China. Three intervention groups were included in this study: a mixed resistance training group with Yi Jin Jing exercises (YR), a resistance training group (RT), and a control group (CG). Participants were assigned to each of the three intervention groups using a randomized grouping method. The intervention was carried out by two trainers with six years of experience in Yi Jin Jing training and two fitness coaches, and six nurses were present throughout the trial.

It is well-established that QCT can exclude measurement errors caused by unstable CT values and can more accurately assess skeletal muscle morphology and structure, showing the degree of muscle edema and steatosis [43]. Two imaging physicians conducted quantitative measurements of skeletal muscle and fat at the third lumbar vertebra. The trial was divided into weekly sessions conducted on Tuesdays, Thursdays, and Saturdays for 24 weeks.

With the consent of the Ethics Committee of Northeast Normal University (approval number: NC2019030702), we conducted a 24-week intervention trial from 1 June to 30 December 2019, and all participants who met the criteria signed an informed consent form. Participants were not required to participate in any other form of physical activity during the trial.

#### 2.2.2. Intervention

In this study, we designed three sets of intervention programs including Yi Jin Jing exercise and resistance training, resistance training, and a control group. At the beginning of each session, all groups underwent a 20 min warm-up. The YR group consisted of 30 min of Yi Jin Jing exercise and 30 min of resistance training. The RT group consisted of 60 min of resistance training. Patients in the CG group were educated on sarcopenia and prevention methods, such as paying attention to increasing dietary protein intake and participating in more physical activities. At the end of each session, we arranged a ten-minute relaxation activity. The following is a detailed intervention programs:

(1) Yi Jin Jing exercise: The training schedule of Yi Jin Jing consisted of two phases of 8 and 16 weeks, respectively. The first phase involved learning the movements: two Yi Jin Jing exercises were practiced in each session. During the second phase, the main goal was to consolidate learning of the exercise, and three Yi Jin Jing exercises were practiced in each session.
(2) Resistance training: Resistance training consists of a total of five exercises which include two lower body exercises with supine elastic band resistance leg lifts and standing elastic band resistance leg raises, and three upper body exercises, including bicep curls, reverse grip curl, and seated pull down. To maximize strength gains the muscle hypertrophy, the training session was divided into three 8-week periods. The purpose of the first phase was to allow participants to learn and master the training movements using low loads (40.0–60.0% of 1 RM) and high repetitions (12–20) for 2–4 training sets. The second stage aimed to induce muscle hypertrophy and further improve muscle mass and reduce the fat content of the skeletal muscle interstitium by gradually increasing the load with a medium load of (60.0–80.0% of 1 RM), 5–12 repetitions, for 2–4 sets. In the third phase, we used a higher load (70.0–85.0% of 1 RM) and reduced the number of repetitions (5–8 repetitions) for 2–4 sets to further optimize muscle strength and increase maximal muscle power. The RT group underwent 4 sets for each movement, whereas the YR group completed 2 sets, with a 2 to 3 min interval between sets.
(3) Control group: The CG group underwent conventional treatment, and the nurse educated the participants on sarcopenia and prevention methods, such as increasing protein intake in the diet and participating in more physical activities.

Requirements for participants: Participants were required to wear comfortable sportswear and sneakers during the training and report any physical discomfort during the training. Training could be terminated at any time.

#### 2.2.3. Assessment of Sarcopenia

We screened all participants according to the screening criteria specified by the Asian Working Group for Sarcopenia (AWGS) [41]. A diagnosis of sarcopenia was established when the following criteria were met:

(1) Handgrip strength: Subjects’ handgrip strength was measured using a calibrated Jamar Hydraulic Hand Dynamometer (model SH5001, Saehan Corp, Masan, Korea, 2017). The handgrip strength test was in a standing position, and the shoulder was aligned with the torso, with the elbow fully extended, with the wrist maintaining a neutral position [44]. Each participant took two handgrip strength tests and the maximum value was used for this study. The handgrip strength of less than 28 kg for men and less than 18 kg for women met the screening criteria.
(2) Physical performance: According to recommendations of AWGS 2016, we used a six-meter gait speed to assess physical performance. Participants stand with both feet on the starting line, and when hear the command “go”, walk along the 6-meter route at normal speed, and walk a few steps past the finish line [45]. The cut-off value of gait speed was 0.8 m/s.
(3) Appendicular skeletal muscle mass (ASM): Appendicular skeletal muscle mass was performed on participants using a multifrequency with eight tactile electrodes (Inbody S10 Biospace, Biospace Co., Ltd., Seoul, Korea). When the ASM was less than 7.0 kg/m${}^{2}$ for men and less than 5.7 kg/m${}^{2}$ for women met the screening criteria.

#### 2.2.4. Assessment of Skeletal Muscle and Fat

The GE Revolution 256-row CT (General Electric Company, Boston, MA, USA, 2015) was used to scan the upper abdomen of the participants once each before and one week after the intervention. Participants were placed in a supine position with both arms raised. GSI scan parameters included a tube voltage 80, 140 kVp transient switching, tube current intelligent adjustment, layer thickness 5 mm, layer spacing 5 mm, and data were automatically imported into the GE ADW 4.7 workstation at the end of the scan. We selected the L3 level in the GE ADW 4.7 workstation and manually outlined the area of interest along the skeletal muscle using the X Section software within the workstation.

##### Measurement of Skeletal Muscle

Skeletal muscle was measured on abdominal CT, the skeletal muscle cross-sectional area at the third lumbar vertebra (L3 SMA, cm${}^{2}$) and skeletal muscle density at the third lumbar vertebra (L3 SMD, HU) were measured using a Hounsfield unit range of −30 to 150 due to its accuracy in reflecting the actual muscle mass and fat volume [46].

##### Measurement of Skeletal Muscle Interstitial Fat

The skeletal muscle interstitial fat area within the area of interest at the third lumbar vertebra (L3 SMFA, cm${}^{2}$) and skeletal muscle interstitial fat density at the third lumbar vertebra (L3 SMFD, HU) were measured before and after the intervention. The quantitative fat measurement threshold was selected from −200 to 0 HU.

##### Measurement of Relative Skeletal Muscle Mass Index

Baumgartner et al. [47] created a relative index to measure muscle development, the relative skeletal muscle mass index (RSMI, kg/m${}^{2}$). RSMI is widely used in diagnosing sarcopenia and is calculated as skeletal muscle mass per square height of the appendage. Therefore, we used RSMI as one of the observation indicators. Multifrequency bioelectrical impedance analysis with eight tactile electrodes (Inbody S10 Biospace, Biospace Co., Ltd., Seoul, Korea) was used to conduct RSMI.

##### Measurement of Muscle Fat Infiltration

A study demonstrated that muscle fat infiltration (MFI, %) leads to a decrease in skeletal muscle mass per unit, triggering muscle atrophy and affecting the development of sarcopenia [48]. Therefore, we calculated the MFI of the participants. The formula is shown in Equation (1):

(1) MFI=SMFA/(SMA+SMFA)×100%,

##### Measurement of Handgrip Strength

Handgrip strength is the preferred indicator for the diagnosis of sarcopenia and is also an indirect indicator of overall muscle strength [49]. Therefore, we assessed the participant’s grip strength before and after the intervention. Participants’ handgrip strength was measured using a calibrated Jamar Hydraulic Hand Dynamometer (model SH5001, Saehan Corp, Masan, Korea, 2017). The handgrip strength test was in a standing position, and the shoulder was aligned with the torso, with the elbow fully extended, with the wrist maintaining a neutral position [44]. Each participant took two handgrip strength tests and the maximum value was used for this study.

#### 2.2.5. Data Analysis

We used 10 classical machine learning classification models and a stacking model to predict whether sarcopenia could be reversed in participants after the intervention. The sex, age, pre-intervention L3 SMA, L3 SMFA, RSMI, handgrip strength, and the three intervention types were used as features of the dataset, and whether the participants were sarcopenic after the intervention was used as a label, with sarcopenic noted as 1 and normalities noted as 0. The accuracy, recall, prediction, F1 score, and area under the curve (AUC) were used to evaluate the performance of classification models. We used a 10-fold cross-validation training strategy, and each model was trained one hundred times, separately. First, we train ten classical machine learning models using LightGBM Classifier (LGBM) [50], Gradient Boosting Classifier (GBC) [51], XGBoost Classifier (XGB) [52], Extra Tree Classifier (ETC) [53], k Neighbors Classifier (KNN) [54], Decision Tree Classifier (DT) [55], Random Forest Classifier (RF) [56], Linear Discriminant Analysis (LDA) [57], Support Vector Classifier (SVC) [58], and Logistic Regression (LR) [59]. Then, we used the three models with the highest average accuracy for stacking. Multiple classifiers developed by various learning algorithms $L_{1},\ldots ,L_{n}$ were combined in this work to accomplish stacking on a single dataset *S*, which consisted of $S_{i}$ = ($x_{i}$, $y_{i}$). Feature vectors are represented by $x_{i}$, whereas classifications are represented by $y_{i}$. First, a set of base classifiers $C_{1}$, $C_{2}$, and $C_{3}$ was generated, where $C_{i}$ = $L_{n}$ (*S*). A meta classifier was learned in the second phase, and it comprised the outputs of the basis classifiers. Each of the base-level learning methods was applied to almost the full dataset as part of a cross-validation approach to create a training set for the meta classifier, leaving one example for testing, as: $\forall i=1,\ldots ,n:\forall k=1,\ldots ,N:C_{k}^{i}=L_{k}\left(S-s_{i}\right)$. Then, we generated predictions for $S_{i}$ using the learned classifiers, as shown in Equation (2):

(2) $${\hat{y}}_{i}^{k}=C_{k}^{i}\left(x_{i}\right);$$

Here, the meta-level dataset consists of examples of the form ((${\hat{y}}_{i}^{1}$, …, ${\hat{y}}_{i}^{n}$), $y_{i}$), where the features are the predictions of the base-level classifiers and the class is the correct class of the example at hand.

We used Shapley additive explanation (SHAP) values to figure out which feature was the most important [60]. SHAP is a game-theoretic explanation for the output of any machine learning model; the SHAP values may be used to quantify how each feature contributes to the model’s prediction, as shown in Equation (3):

(3) $$\phi_{j}=\sum_{S_{F}\subseteq F\backslash \left\{j\right\}}\frac{|S_{F}\left|!(|F|-\right|S_{F}\left|-1\right)!}{|F|!}\left[f_{S_{F}\cup \left\{j\right\}}\left(x_{S_{F}\cup \left\{j\right\}}\right)-f_{S_{F}}\left(x_{S_{F}}\right)\right],$$

where *x* is the values of the input features, *j* is a certain feature (out of total features *F*), $S_{F}$ indicates all possible subsets without feature *j*, and $|S_{F}|$ is the dimension of $S_{F}$. To compute this effect, a model $f_{S_{F}\cup \left\{j\right\}}$ was trained with feature *j* present, and another model $F_{S_{F}}$ was trained with feature *j* withheld. In this study, the SHAP “TreeExplainer” algorithm was used to determine the most important feature in predicting whether the post-intervention participant was a patient with sarcopenia. Python 3.7.1 was used to conduct all data analysis in this study.

#### 2.2.6. Statistical Analysis

The experimental data were statistically analyzed using SPSS 25.0 software, and the S–W (Shapiro–Wilk) test was used to test the distribution of the data. For data that conformed to a normal distribution, the variables between groups were analyzed for differences between groups before the experiment using one-way ANOVA, Bonferroni post hoc test, and chi-square test. Intergroup and time interactions for each variable were tested using two-way repeated-measures ANOVA, and simple effects analysis was performed for variables with interactions. Continuous variables are expressed as mean ± SD and categorical variables are expressed as numerical values. *p* < 0.05 indicates statistical significance.

## 3. Results

We recruited a total of 132 participants, and after screening based on inclusion and exclusion criteria, 104 participants (51 females and 53 males) met the criteria. Finally, 90 participants (47 females and 43 males) were included in this study. The causes for not completing the training were dropout (*n* = 8) and illness-related termination (*n* = 6). Table 1 shows the demographic information of the participants at baseline.

**Figure 3:**
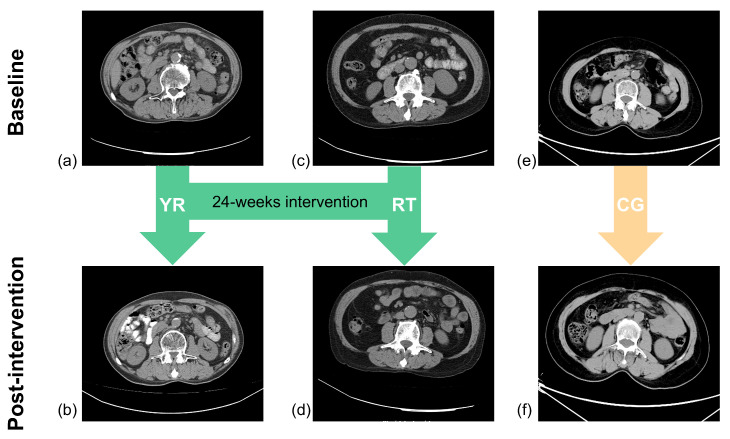
This figure shows the QCT scans of the participants before and after the intervention. It shows the changes in L3 SMA and L3 SMFA at baseline and after the intervention in older patients with sarcopenia. (**a**) Pre-intervention YR group; (**c**) pre-intervention RT group; (**e**) pre-intervention CG group; (**b**) post-intervention YR group; (**d**) post-intervention RT group; (**f**) post-intervention CG group.

At the end of the intervention, the researchers reassessed the subjects for sarcopenia. The results showed that our intervention protocol resulted in the reversal of sarcopenia in 27.8% (*n* = 25) of participants, including 52.0% (*n* = 13) in the YR group and 48.0% (*n* = 12) in the RT group. The QCT results of the participants are shown in Figure 3.

**Figure 4:**
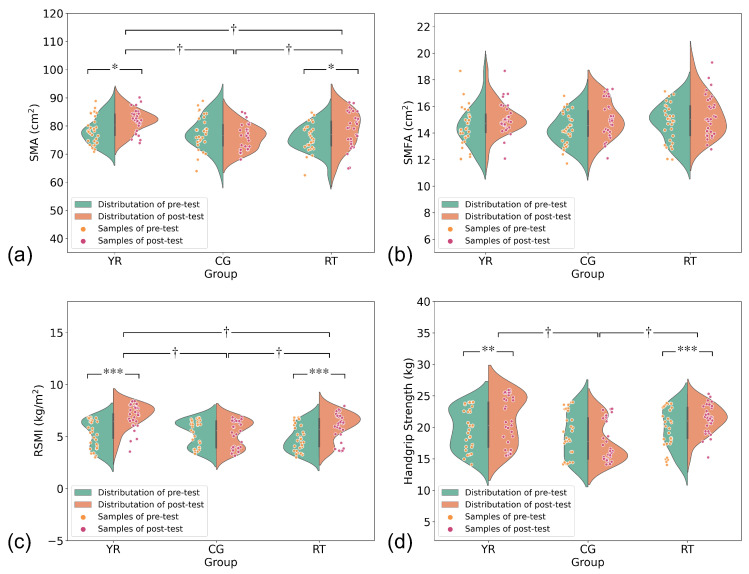
A scatter plot with a rotated kernel density plot on each side showing the changes in L3 SMA, L3 SMFA, RSMI, and handgrip strength in older patients with sarcopenia at baseline and after the intervention. (**a**) L3 SMA, (**b**) L3 SMFA, (**c**) RSMI; (**d**) handgrip strength. YR indicates Yi Jin Jing and resistance training group; RT indicates resistance training group; CG indicates control group. * *p* < 0.05, ** *p* < 0.01, *** *p* < 0.001 the significance of intragroup differences, † the significance difference among groups (*p* < 0.05).

In addition, Figure 4 shows the main results, and Table 2 shows the two-way repeated-measures ANOVA results for L3 SMA, L3 SMD, L3 SMFA, L3 SMFD, RSMI, MFI, and handgrip strength. The two-way repeated-measures ANOVA showed that the group effects of L3 SMA, L3 SMD, L3 SMFA, L3 SMFD, RSMI, MFI, and handgrip strength were not significant at baseline. There was a significant group × time interaction for L3 SMA (*p* < 0.05), RSMI (*p* < 0.05), MFI (*p* < 0.05) and handgrip strength (*p* < 0.05). Participants in the YR and RT groups showed significant improvements in L3 SMA, RSMI and handgrip strength after the intervention. Post hoc tests showed significantly better L3 SMA and RSMI after the intervention in the YR group than in the RT group (*p* < 0.05) and CG group (*p* < 0.05). There was no significant group × time interaction for L3 SMD, L3 SMFA, and L3 SMFD.

We used whether the participants were sarcopenic after the intervention as a label, and sex, age, L3 SMA, L3 SMFA, RSMI, handgrip strength, and the three intervention types of the subjects before the intervention as features to train the model for predicting whether sarcopenia was reversed after the intervention. Table 3 shows the performance of the models. Then, the dataset was input into the first layer model that was built using the three classification models Linear Discriminant Analysis, LGBM Classifier, and Logistic Regression to create the super features. The super features and label were then input as data to the second part of the training, which used the Logistic Regression.

The results demonstrated that our stacking model produced the greatest accuracy (85.7 ± 10.6%) and the highest F1 score (75.3 ± 11.5%). However, the LightGBM Classifier demonstrated the best precision score (78.7 ± 10.8%), and the LDA had the best recall score (77.3 ± 12.9%). In addition, we evaluated the surrogate dataset’s accuracy (60.3 ± 10.3%) and found that stacking performed much better than chance.

**Figure 5:**
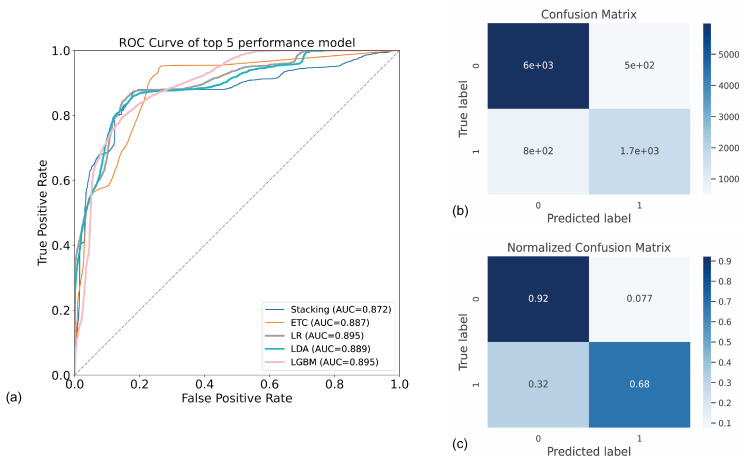
The ROC curve and the confusion matrix of stacking model. (**a**) The ROC curve of the top-5 performance models. ETC indicates Extra Tree Classifier; LR indicates Logistic Regression; LDA indicates Linear Discriminant Analysis; LGBM indicates LightGBM Classifier. (**b**) Confusion matrix; (**c**) normalized confusion matrix. The figure shows the model’s performance in identifying whether a participant has sarcopenia.

In addition, Figure 5a depicts the model receiver operating characteristic (ROC) curve used to assess model performance; the false-positive rate is shown by the horizontal coordinate; the vertical axis represents the actual positive rate; the horizontal coordinate indicates the false-positive rate; the vertical coordinate indicates the genuine positive rate; the highest AUC value of the Logistic Regression and LGBM Classifier models was 0.895. The confusion matrix’s output represents the model’s sarcopenia prediction, and this study showed that both the confusion matrix and the normalized confusion matrix exhibited a good prediction performance. The predicted categories were represented by each row of the confusion matrix in Figure 5b, and each column represents the true label, while the normalized confusion matrix, which represents the average accuracy of the model’s predictions for each category (92.0% and 68%, respectively), is shown in Figure 5c.

**Figure 6:**
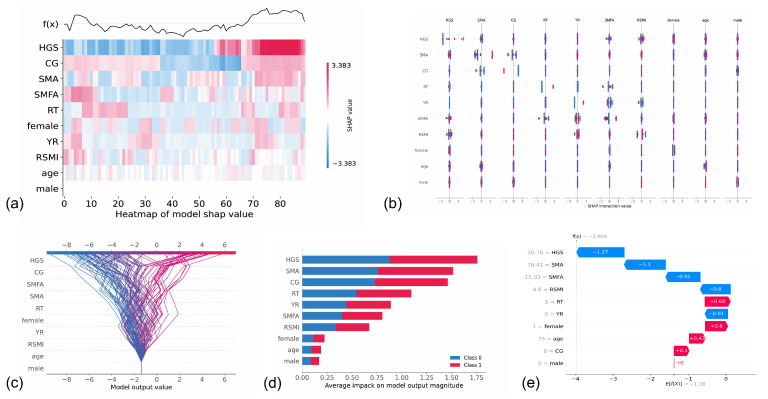
A summary plot of the SHAP values of all features of the stacking model. The y-axis indicates the different features. HGS: handgrip strength. (**a**) illustrates heat map of various feature SHAP values, where the *x*-axis represents the sample sequence, red represents positive impact, and blue represents negative influence; the deeper the hue, the higher the degree of effect. f(x) denotes the output (before activation function). If the output is positive, the graphic illustrates that the most essential characteristic has a positive influence. (**b**) represents the interaction SHAP values of several characteristics, demonstrating the interaction effects between them. (**c**) illustrates the cumulative process for each sample and each feature of the model, and the *x*-axis indicates the final predicted value. (**d**) denotes the average absolute SHAP value of different features; the *x*-axis denotes the average SHAP value. The handgrip strength contributed the most to predicting whether the post-intervention participant was a patient with sarcopenia. (**e**) illustrates the influence of features in a single sample, the *x*-axis represents the SHAP value. The handgrip strength contributed the most positive effect, and the RT had the most negative effect in predicting whether the post-intervention participant was a patient with sarcopenia.

Figure 6 displays the contribution of each feature based on the SHAP value. The SHAP value indicates how much each feature contributes to the model’s performance. The results showed that female (gender), L3 SMA, and CG (the intervention type) yielded the largest contribution among the intervention protocols, suggesting that these are the most crucial factors for predicting the results. Furthermore, most characteristics could interact with one another, indicating the complex interplay mechanism (Figure 6b). By comparing the performance of all feature combinations, the best combination of features could be identified to improve the model’s performance.

## 4. Discussion

As far as we know, there has never been a previous study proposing a hybrid exercise strategy incorporating RET and CTE for older adults with sarcopenia. Moreover, artificial intelligence was used to predict whether the intervention could reverse sarcopenia in older adults. This study showed that YR and RT groups demonstrated different degrees of physical improvements in L3 SMA, RSMI, and handgrip strength at 24 weeks. The YR group demonstrated the most significant improvement in L3 SMA and RSMI. Additionally, the hybrid exercise program consisting of YR and RT demonstrated optimal results in reversing sarcopenia, with 27.8% of older adults recovering from sarcopenia.

Meanwhile, we innovatively introduced XAI for sarcopenia prediction in an external sample of older adults from China, which yielded an average accuracy of 85.7%. Furthermore, our proposed stacking model differentiated the reversing of sarcopenia and explained the feature contribution based on the SHAP value. This prediction method is a substantial improvement compared with previously published conventional methods.

**Figure 7:**
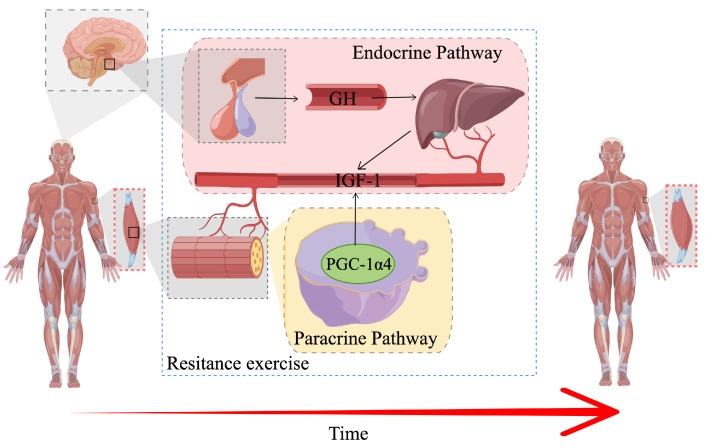
Schematic of the Major Signaling Pathways Involved in the Control of Skeletal Muscle Hypertrophy.

Herein, we found that after 24 weeks of YR and RT interventions, both groups of older patients with sarcopenia exhibited an increase in L3 SMA, RSMI, and handgrip strength and patients in the YR group experienced a significant improvement in L3 SMA and RSMI compared to the RT (*p* < 0.05) and CG (*p* < 0.05) groups. These results are consistent with findings of a previous work [26], where RET intervention could improve the physical condition and reverse sarcopenia. It is widely acknowledged that for sarcopenia in older adults, loss of muscle mass is due to both a loss of myofibers and a decrease in myofiber cross-sectional area, especially in fast contracting myofibers [61]. Therefore, the improvement in SMA may partly be explained by increased IGF1 expression, which can inhibit atrophy and promote hypertrophy. It has been demonstrated that mechanical load contraction during resistance exercise has a series of effects that positively contribute to the net accumulation of intracellular proteins, thereby promoting the expansion of muscle fibers and the remodeling of the extracellular matrix [62]. Specifically, RET promotes PGC-1α expression in exercised muscle, which may induce IGF1 and represses myostatin, inducing robust skeletal muscle hypertrophy [63]; on the other hand, mechanical loading promotes GH expression. GH is reportedly a powerful regulator of post-natal bone growth, whose effects are mainly mediated by the GH/IGF-1 axis and increased IGF1 gene expression, as shown in Figure 7.

**Figure 8:**
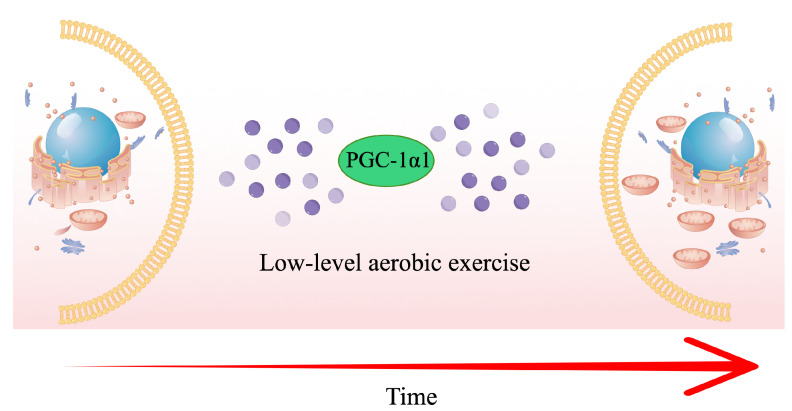
Schematic of the Major Signaling Pathways Involved in the Control of Mitochondrial Biogenesis.

Importantly, we found that the YR group experienced a more significant improvement than the RT group in L3 SMA (*p* < 0.05) and RSMI (*p* < 0.05) after 24 weeks. The results are consistent with previous studies that reported that hybrid exercise might enhance the effect on muscles [64,65]. For centuries, CTE, a series of mind–body low-level aerobic exercises including Yi Jin Jing, Taichi, Baduanjin, etc., in order to prevent and treat diseases, has been widely used in China. Since the mechanism of Yi Jin Jing remains largely unclear, this limitation means that the effectiveness of the hybrid exercise program needs to be interpreted cautiously. It is widely thought that as a low-level aerobic exercise, regular exercise (aerobic training) has many positive effects on physical fitness, such as improving endurance, insulin sensitivity, and fat metabolism. This effect is associated mainly with a marked increase in skeletal muscle mitochondrial volume/density with the activation of the PGC-1α/FNDC5/UCP1 signaling pathway and upregulation of PGC-1α [66]. In addition to leading to an increase in the number and activity of mitochondrial enzymes, it also leads to an increase in fat oxidation in muscles at rest and at low intensities [67,68,69,70], as shown in Figure 8.

Moreover, based on traditional Chinese medicine’s theoretical principles, clinical rehabilitation emphasizes the importance of posture, meditation, and breathing coordination. Slow, relaxing, and systematic movements are common characteristics of these movements, making them suitable for physically weak patients. Therefore, it is important to maintain calmness and stillness of mind, as it helps to correct poor body posture, adjust wrong breathing patterns, and maintain body movement. Several natural self-regulatory/self-healing mechanisms, once activated, allow for a balanced release of endogenous neurohormones [71,72]. Finally, as above-mentioned, the benefits during RET include an increased number of muscle fibers and cross-sectional area, which accounts for the effectiveness of the hybrid exercise program. Therefore, integrating RET and Yi Jin Jing yielded better outcomes than other strategies. Additionally, we found that after the exercise intervention, the skeletal muscle interstitial fat area at the L3 level exhibited a rising trend in all three groups, although no statistical significance was found (*p* > 0.05). In contrast, there was no significant difference in the fat area of skeletal muscle interstitial space at the L3 vertebral level between the three groups after the intervention (*p* > 0.05). It is well-established that the interstitial fat content gradually increases with age in the older adults [73,74]. In the present study, both interventions (hybrid program and RET) could not reverse interstitial fat accumulation, contrary to previous studies that suggested that aerobic exercise and RT helped prevent age-related interstitial fat accumulation in the older adults [75]. This finding may be because the intensity of aerobic exercise was not sufficient.

Perhaps the most clinically relevant finding is that a total of 27.8% (*n* = 25) of participants reversed their sarcopenia, including 52.0% (*n* = 13) of the YR group and 48.0% (*n* = 12) of the RT group. To the best of our knowledge, this is the first study to evaluate sarcopenia recovery based on an exercise intervention, and our finding broadly supports the work of other studies in a similar area linking the recovery from geriatric disease with hybrid exercise intervention [64]. As defined from AWGS [41], sarcopenia exhibits low hand-grip strength, low physical performance, and low skeletal muscle mass. Therefore, this result may be explained as above mentioned, the hybrid exercise program showed better improvement in L3 SMA and RSMI which are crucial indicators of sarcopenia; on the other hand, an exercise program can effectively improve physical fitness [4,26,64] and reduce frailty among older adults [64], these factors may explain the relatively good correlation between exercise program and sarcopenia.

Although promising results were observed, they should be interpreted with caution due to significant limitations and shortcomings in our study. First of all, given that previous studies have established the effectiveness of Yi Jin Jing on skeletal muscle, no Yi Jin Jing group was set in our study. In addition, although the hybrid exercise program was able to improve SMA and RSMI in older patients with sarcopenia, it still has significant limitations in terms of skeletal muscle fat. Finally, we suggest that sarcopenia in older adults may be closely linked to the reversal of sarcopenia. We intend to incorporate the data to build a more comprehensive system with different features to provide more accurate medical assistance to this patient population based on the association between sarcopenia reversal, the starting condition of sarcopenia in older adults, and the intervention program.

## 5. Conclusions

Our findings indicate that a hybrid exercise program of RCE and Yi Jin Jing can effectively improve muscle mass and reverse sarcopenia in older adults. Additionally, our established stacking model could predict sarcopenia in older adults with good accuracy.

## Figures and Tables

**Table 1:** Results of demographic characteristics of the participants at baseline.

Items	YR ^1^ (*n* = 30)	RT ^2^ (*n* = 30)	CG ^3^ (*n* = 30)	*p*-Value
Sex (male/female)	14/16	13/17	16/14	0.745
Age (years)	66.70 ± 4.10	66.87 ± 3.84	65.42 ± 3.97	0.301
Body mass (cm)	166.13 ± 7.67	168.11 ± 8.58	167.14 ± 8.35	0.217
Stature (kg)	63.13 ± 6.19	64.37 ± 7.16	61.05 ± 7.87	0.417
BMI (kg/m${}^{2}$)	22.76 ± 2.19	22.80 ± 3.18	21.93 ± 2.86	0.870

^1^ Yi Jin Jing exercise and resistance training group; ^2^ resistance training group; ^3^ control group.

**Table 2:** Results of a two-way repeated-measures ANOVA at baseline and 24 weeks for each group.

Parameters	YR ^1^ (*n* = 30)		RT ^2^ (*n* = 30)		CG ^3^ (*n* = 30)		Group × Time ^#^
	Baseline	24 Weeks	Baseline	24 Weeks	Baseline	24 Weeks	*p*-Value
SMA (cm${}^{2}$)	78.39 ± 4.71	81.92 ± 3.99 ^†,^*	75.79 ± 4.77	78.64 ± 6.36 ^†,^*	77.63 ± 5.81	77.06 ± 4.53 ^†^	<0.05
SMD (HU)	32.53 ± 3.14	34.25 ± 3.15 ^†,^**	32.64 ± 3.03	34.72 ± 2.80 ^†,^**	32.69 ± 3.72	32.44 ± 3.31 ^†^	>0.05
SMFA (cm${}^{2}$)	14.43 ± 1.46	15.11 ± 1.22	14.73 ± 1.43	15.30 ± 1.58	14.28 ± 1.24	15.03 ± 1.39	>0.05
SMFD (HU)	−64.73 ± 5.90	−65.05 ± 5.83	−64.01 ± 5.43	−64.48 ± 4.71	−64.21 ± 5.69	−64.68 ± 6.11	>0.05
RSMI (kg/m${}^{2}$)	5.25 ± 1.32	6.98 ± 1.18 ^†,^***	4.84 ± 1.23	6.05 ± 1.30 ^†,^***	5.32 ± 1.21	5.24 ± 1.28 ^†^	<0.05
MFI (%)	15.57 ± 1.53	15.58 ± 1.24	16.30 ± 1.61	16.35 ± 1.93	15.60 ± 1.82	16.52 ± 1.52 **	<0.05
HGS (kg)	19.70 ± 3.22	21.21 ± 3.80 ^†,^**	19.73 ± 3.13	21.35 ± 2.29 ^†,^***	18.72 ± 3.48	17.67 ± 3.10 ^†^	<0.05

All data are expressed as Means ± SD; ^#^ analysis of two-way repeated-measures ANOVA; ^†^ significant difference between groups (*p* < 0.05); * *p* < 0.05, ** *p* < 0.01, *** *p* < 0.001 significant difference between baseline and post-intervention. ^1^ Yi Jin Jing exercise and resistance training group; ^2^ resistance training group; ^3^ control group.

**Table 3:** Model performance evaluation results.

Models	Accuracy	Precision	Recall	F1
SVC ^1^ (%)	72.2 ± 11.6	60.2 ± 11.4	64.8 ± 12.3	65.2 ± 11.1
KNN Classifier ^2^ (%)	79.1 ± 11.4	61.3 ± 11.6	63.8 ± 12.1	68.6 ± 12.1
XGB Classifier ^3^ (%)	79.6 ± 11.9	64.0 ± 12.7	61.1 ± 11.3	60.1 ± 12.5
Decision Tree (%)	80.4 ± 12.5	66.1 ± 11.4	66.0 ± 11.3	63.4 ± 15.8
Extra Tree Classifier (%)	81.1 ± 11.2	67.6 ± 13.4	61.3 ± 10.5	60.2 ± 14.2
GDB Classifier ^4^ (%)	82.0 ± 11.7	70.5 ± 11.4	61.4 ± 11.2	63.0 ± 16.6
RF Classifier ^5^ (%)	82.9 ± 11.5	71.8 ± 11.9	61.9 ± 10.1	63.4 ± 13.1
Logistic Regression (%)	84.0 ± 10.9	74.6 ± 12.1	69.7 ± 11.7	68.5 ± 14.1
LGBM Classifier ^6^ (%)	85.0 ± 11.3	**78.7 ± 10.8**	64.8 ± 10.4	67.1 ± 10.8
LDA ^7^ (%)	85.4 ± 11.1	75.1 ± 11.6	**77.3 ± 12.9**	73.4 ± 12.8
**Stacking (%)**	**85.7 ± 10.6**	78.2 ± 13.2	74.5 ± 15.9	**75.3 ± 11.5**

^1^ Support Vector Classification; ^2^ K Neighbors Classifier; ^3^ XGBoosting Classifier; ^4^ Gradient Boosting Classifier; ^5^ Random Forest Classifier; ^6^ LightGBM Classifier; ^7^ Linear Discriminant Analysis. We examined the performance of several models, and the stacking model performed the best in accuracy and F1 score. As the first layer, the stacking model used Logistic Regression, Linear Discriminant Analysis, and LightGBM Classifier, and as the second layer, it used Logistic Regression.

## Data Availability

The datasets used and/or analyzed during the current study are available from the corresponding author upon reasonable request.
